# “Spend your spoons wisely”: Conceptualizations of time, energy and aging invisibly with Crohn’s Disease

**DOI:** 10.1093/geroni/igab046.2305

**Published:** 2021-12-17

**Authors:** Ginny Natale, Manacy Pai

**Affiliations:** 1 Stony Brook University, Stony Brook, New York, United States; 2 Kent State University, Kent, Ohio, United States

## Abstract

An increasing number of people with chronic medical disabilities are living longer and into old age due to the growing medical and technological advancements over the past half century. We used grounded theory to examine the lived experience of aging “with” a disability in a non-elderly population. On average, participants were 37 years of age at the time of interview. The average time since diagnosis was 17 years and ranged from 3 to 34 years. Many worked full-time outside of the home and some held advanced or graduate degrees. Of the 35 participants interviewed, three-quarters expressed worries about the future and aging, specifically related to physical limitations of having CD. The other 25% talked about learning to accept the diagnosis and ‘moving forward’ with their life as they age. All participants described the difficulties of fatigue and energy limitations. Planning of life was limited to 24 hours — a direct consequence of functional limitations of a relapsing-remitting disease. The most prominent theme that emerged from participants’ narratives to explain aging invisibly with a chronic illness was quantifying energy into ‘spoons’, a way of measuring the stock of their energy on any given day. These findings translate into important insights into the process of aging for those who live and age “with” Crohn’s as their everyday lives are immersed in managing the varying whims of this illness.

